# Interactions Between Bovine Respiratory Syncytial Virus and Cattle: Aspects of Pathogenesis and Immunity

**DOI:** 10.3390/v16111753

**Published:** 2024-11-08

**Authors:** Lariane da Silva Barcelos, Alexandra K. Ford, Matheus Iuri Frühauf, Nadalin Yandra Botton, Geferson Fischer, Mayara Fernanda Maggioli

**Affiliations:** 1 Department of Veterinary Pathobiology, Oklahoma State University, Stillwater, OK 74078, USA; ldasilv@okstate.edu (L.d.S.B.);; 2 Laboratory of Virology and Immunology, Veterinary College, Universidade Federal de Pelotas, Capão do Leão, Rio Grande do Sul 96010, Brazil; matheus.fruhauf@outlook.com (M.I.F.); nadalinyb@gmail.com (N.Y.B.); geferson@ufpel.edu.br (G.F.)

**Keywords:** immunology, innate immunity, signaling pathways

## Abstract

Bovine respiratory syncytial virus (BRSV) is a major respiratory pathogen in cattle and is relevant to the livestock industry worldwide. BRSV is most severe in young calves and is often associated with stressful management events. The disease is responsible for economic losses due to lower productivity, morbidity, mortality, and prevention and treatment costs. As members of the same genus, bovine and human RSV share a high degree of homology and are similar in terms of their genomes, transmission, clinical signs, and epidemiology. This overlap presents an opportunity for One Health approaches and translational studies, with dual benefits; however, there is still a relative lack of studies focused on BRSV, and the continued search for improved prophylaxis highlights the need for a deeper understanding of its immunological features. BRSV employs different host-immunity-escaping mechanisms that interfere with effective long-term memory responses to current vaccines and natural infections. This review presents an updated description of BRSV’s immunity processes, such as the PRRs and signaling pathways involved in BRSV infection, aspects of its pathogeny, and the evading mechanisms developed by the virus to thwart the immune response.

## 1. Introduction

Bovine respiratory syncytial virus (BRSV) belongs to the *Pneumoviridae* family in the *Orthopneumovirus* genus and Mononegavirales order, a group of enveloped negative-sense RNA (ssRNA^−^) viruses [1,2]. Formerly, BRSV was classified as *Paramyxoviridae*, but in 2015, the *Pneumoviridae* family was created with BRSV belonging to this family. BRSV was renamed as *Bovine orthopneumovirus* in 2016. In 2022, BRSV was then renamed *Orthopneumovirus bovis* [2]. We will adhere to the historical terminology to maintain consistency with the previous literature. First described in Europe [3], BRSV was reported in the USA in 1974 [4], and to this day, this agent remains as a relevant respiratory pathogen worldwide [5,6,7,8], acting either as the sole agent involved in respiratory disease in young calves or involved in bovine respiratory disease (BRD) in cattle. BRD is a common illness in young cattle and the most important infectious cause of economic burden to the feedlot industry. The disease is complex and multifactorial, involving a combination of factors such as viral and bacterial pathogens, stress factors (e.g., weaning, transportation, and the commingling of cattle from different sources), and environmental conditions (e.g., dust and temperature fluctuations). BRSV increases the susceptibility of calves to secondary infections and allows for the colonization of the lower respiratory tract by bacteria [9,10,11]. Fatalities, reduced production, prevention, and treatment costs contribute to associated economic losses. Still, more animals displaying lung lesions at slaughter are reported than the number of clinical BRD cases identified in herds, indicating that losses might be underestimated [12]. Respiratory diseases are also one of the leading causes of antimicrobial use in cattle, with consequences for animals, public health, and the environment [13,14]. Young calves are the most susceptible to BRSV, but the generated immunity is short-lived, and reinfections occur even in older animals, maintaining the virus’s circulation [5,15,16].

## 2. BRSV Genome and Structure

**Figure 1:**
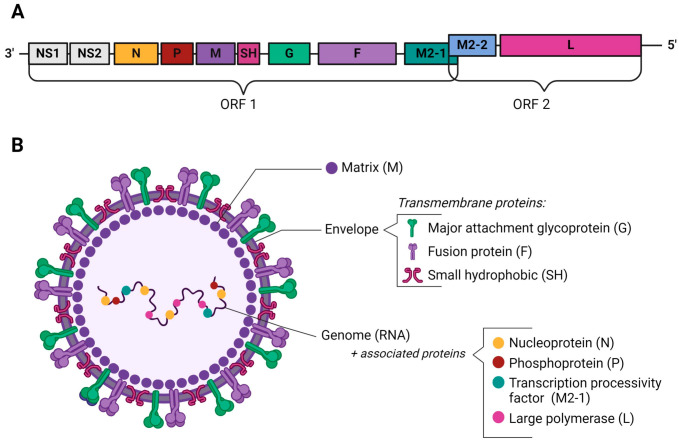
Schematic representation of BRSV genome organization and virion. (**A**) The BRSV genome’s organization is shown—the genome encodes 11 proteins from its ten genes. The M2 gene encodes the M2-1 and M2-2 proteins. (**B**) The circular morphology of the virion is shown. The major attachment (G), fusion (F), and the small hydrophobic (SH) glycoproteins are embedded in the viral membrane. A matrix (M) protein layer lies underneath the viral membrane, giving the virion its overall scaffold and shape. The M2-1 protein interacts with the M and N proteins. The large polymerase subunit (L) and the phosphoprotein polymerase cofactor (P) are also associated with N and the genome. Abbreviations: NS1 and NS2 = nonstructural proteins 1 and 2; N = nucleoprotein; P = phosphoprotein; M = matrix protein; SH = small hydrophobic protein; G = major attachment glycoprotein; F = fusion protein; M2 = matrix protein 2; L = large polymerase protein; ORF1 and ORF2 = open reading frames 1 and 2. Figure created using BioRender (BioRender.com/a08l711).

The BRSV genome consists of an ssRNA^−^ 15.1 kb in length, encoding ten genes containing two overlapping open reading frames (ORFs), resulting in the production of 11 proteins (Figure 1A). The genome sequence from the three prime end to the five prime end includes two nonstructural (NS) genes (NS1 and NS2), followed by nucleoprotein (N) (also called nucleocapsid protein), phosphoprotein (P), matrix protein (M), small hydrophobic protein (SH), fusion glycoprotein (F), major attachment glycoprotein (G), matrix 2 (M2), and large polymerase protein (L). The M2 gene encodes two separate proteins, M2-1 and M2-2, from overlapping ORFs (Figure 1A) [17].

Virions may be spherical but typically present highly filamentous or pleomorphic structures measuring around 200 nm in diameter. The viral particles are encased in a lipid membrane about 7–15 nm thick, which is host-derived. This membrane is adorned with cube-shaped projections forming well-defined bridges between virus particles, creating a unique network structure [18,19,20]. Three viral transmembrane surface glycoproteins are associated with the envelope: G, F, and the SH proteins, while the M protein is located beneath the envelope (Figure 1B). The lipid membrane encloses the nucleocapsid nucleoprotein core, with the M protein forming an outer protein shell around the nucleocapsid [21]. The nucleocapsid is tightly associated with the ssRNA and comprises four viral proteins: N, P, L, and the transcription processivity factor (M2-1) [20,22]. The N protein is responsible for the structure that protects the genome (Figure 1B). The M2-1 and M2-2 proteins, along with the L protein, which acts as the RNA-dependent RNA polymerase (RdRp), are all involved in transcription and genome replication [22].

## 3. Viral Replication

**Figure 2:**
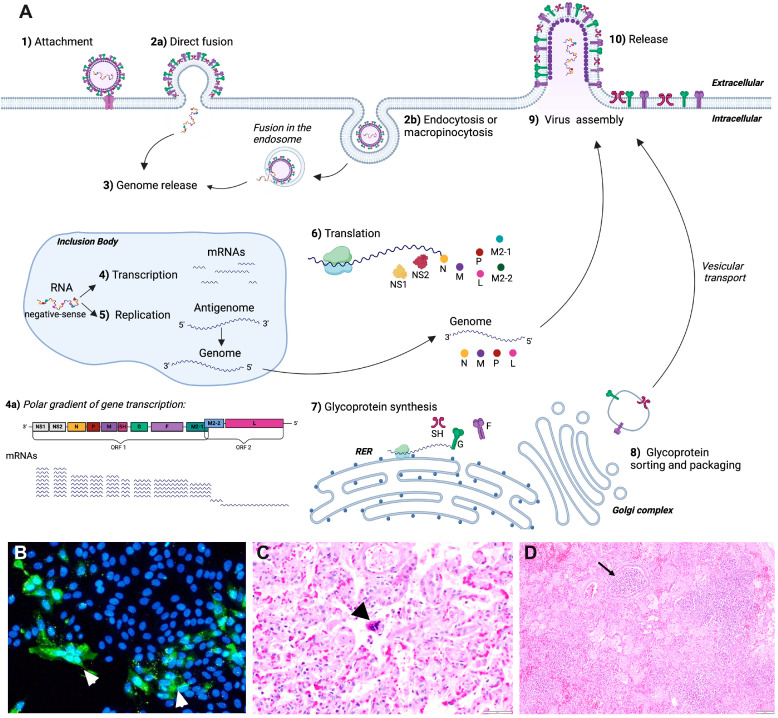
Schematic representation of BRSV replication, images of its cytopathic effect in cell culture, and histological changes in an infected lung. (**A**) Viral entry and replication cycle. (**1**) Viral attachment and viral entry through (**2a**) direct fusion to the host cell membrane mediated by F;or (**2b**) through virions that are internalized via macropinocytosis and clathrin- or caveolin-mediated endocytosis, with fusion taking place in an endosome. (**3**) Upon fusion, the genome is released in the cytoplasm. (**4**) Transcription of mRNAs. (**4a**): Transcription occurs in an obligatorily sequential, polarized manner to generate 10 sub-genomic mRNAs following a gradient in relation to the order in which the genes appear in the ssRNA (genes located closer to the 3′ end of the ssRNA molecule are transcribed at higher levels than genes located towards the 5′ end). (**5**) The antigenome is replicated for progeny generation. (**6**) mRNA from step (**4**) is translated into viral proteins, except for G, F, and SH. (**7**) Glycoproteins G, F, and SH are synthesized in the rough endoplasmic reticulum (RER). (**8**) Glycoproteins G, F, and SH undergo maturation, modification, and packaging in the Golgi complex and are transported in vesicles to the cell membrane. (**9**) Viral assembly: The N protein binds to the newly synthesized viral RNA genome, forming a ribonucleoprotein (RNP) complex, which also includes other genome-associated proteins (P, L, and M2-1). The matrix (M) protein interacts with the RNP complex and assists its transport to the plasma membrane, where assembly occurs. The M protein bridges the RNP complex with the inner surface of the cell membrane, where the G, F, and SH proteins are embedded. (**10**) Newly formed particles bud off or stay associated with the host cell membrane. (**B**) Immunofluorescence staining of BRSV (green) in bovine turbinate infected cells displaying characteristic syncytia formation (white arrows). Cells were infected with BRSV (375) (MOI: ~0.5) and staining was performed 48 h post-infection using 0.01% BRSV anti-serum (VMRD^TM^, Pullman, WA, USA) and 0.4% Alexa Fluor 488 (SouthernBiotech^TM^ Birmingham, AL, USA). Cell nuclei (blue) were counterstained with DAPI (4′,6-diamidino-2-phenylindole). (**C**) Bronchioles and alveolar spaces contain small to moderate amounts of edema, necrotic cellular debris, foamy macrophages, and fibrin. Rare syncytial cells are within bronchioles or alveolar spaces (arrowhead) (40×, H&E). (**D**) Histological findings in a naturally infected calf showing bronchointerstitial pneumonia associated with BRSV. This animal also tested positive for *Histophilus somni*. Alveoli and bronchioles contain degenerate neutrophils, foamy macrophages, edema, and fibrin. Alveoli in most severely affected areas are indistinct. Bronchioles contain abundant cellular exudate (arrow) (10×, H&E). (Image (**B**) is courtesy of Dr. Mayara Maggioli; images (**C**,**D**) are courtesy of Dr. Alexandra K. Ford). Abbreviations: NS1 and NS2 = nonstructural proteins 1 and 2; N = nucleoprotein; P = phosphoprotein; M = matrix protein; SH = small hydrophobic protein; G = major attachment glycoprotein; F = fusion protein; M2 = matrix protein 2; L = large polymerase protein; mRNA = messenger RNA; RER = rough endoplasmic reticulum. Figure 2A was created using BioRender (BioRender.com/e26l126).

Viral entry and replication are initiated by the interaction of the G protein and surface glycosaminoglycans (GAGs) on epithelial respiratory cells (Figure 2A) [23,24], followed by the fusion to the host cell mediated by the F protein [22,25,26]. Alternatively, pneumoviruses’ fusion to the cell membrane may require an internalization mechanism, such as macropinocytosis and clathrin- or caveolin-mediated endocytosis, with the fusion taking place in an endosome upon the proteolytic activation of the F protein (Figure 2A [27,28,29]; reviewed by [30]). Although G is the primary attachment protein, the F protein also has an attachment function, and its expression is sufficient to mediate infection in cell lines but results in an attenuated profile in vivo [31,32,33]. The fusion process is initiated when the F protein binds to a specific receptor on respiratory epithelial cells. This interaction triggers a conformational change in the F protein, converting it from its pre-fusion form to a post-fusion conformation, allowing the fusion peptide of the F protein to be inserted into the plasma membrane and a stable fusion pore formation [34]. As a result, the viral envelope and the host cell membrane merge, and the viral genome can enter the host cell (Figure 2A) [34,35,36]. While studies confirming the cellular receptors for BRSV fusion are still lacking, human RSV F protein binds to nucleolin, which functions as a cell receptor for the virus [37], with several co-receptors also identified, including C-type lectins [38], intercellular adhesion molecule 1 (ICAM-1) [39], epidermal growth factor receptor (EGFR) [40], insulin-like growth factor 1 receptor (IGF1R) [41], and toll-like receptor 4 (TLR-4), which also presents recognition activity related to the immunity against BRSV infection [42,43].

The viral RNA and associated nucleoproteins (N, P, and L) are released into the host cytoplasm upon fusion. Transcription takes place following a three′ to five′ gradient (Figure 2A). The polymerase transcribes each gene, resulting in sub-genomic mRNAs (with a five′ to three′ orientation), which are in turn translated into viral proteins by the host cell machinery. The viral genome is replicated for progeny generation by transcription of the positive-sense antigenome (with a five′ to three′ orientation) by the polymerase, later generating new negative-sense full-genome copies. Viral assembly and maturation occur at the plasma membrane [44,45]. Recent evidence shows that some initial steps of RSV virions’ assembly may alternatively happen within the cytoplasm before the complexes reach the plasma membrane [46,47]. Upon assembly, new virions bud out or stay closely associated with the host cell membrane (Figure 2A), leading to the formation of syncytia [22,48,49].

The F protein is primarily responsible for syncytium formation, the characteristic fusion of infected cells, from which BRSV derives its name [25]. While the binding of the virion to a host cell leads to viral entry [30,34,50], during viral replication, the F protein is expressed on the host cell plasma membrane, mediating cell-to-cell fusion and multinucleated syncytium formation [51]. This cytopathic characteristic is a feature of infection and occurs both in cell lines (Figure 2B) and in vivo (Figure 2C). In infected animals, syncytia are observed mainly as large multinucleated cells formed by infected respiratory epithelial cells (Figure 2C). Syncytium formation may contribute to the pathogenesis of BRSV and is thought to influence viral replication kinetics and viral spread along neighboring cells while minimizing virus exposure to extracellular components of the immune system [32,52,53]. Additionally, due to its critical role in the viral infection process, the F protein is a target for vaccine development [54,55,56,57]. Vaccines that elicit a strong immune response against the F protein may protect cattle from BRSV infections. Still, strong antibodies directed against post-F conformation have been implicated in vaccine-enhanced disease with disastrous consequences in humans [58] and contradictory evidence in cattle [59,60]. Similarly, antiviral drugs that inhibit the function of the F protein could potentially prevent the virus from entering host cells, thereby blocking infection [61,62].

## 4. Common Pathological Findings

BRSV infection often triggers increased mucus production and inflammation, contributing to exudate accumulation in the bronchioles and alveoli. Bronchiolitis, characterized by inflammation, necrosis, and obstruction of the bronchioles, leads to airway constriction, impaired airflow, and respiratory distress, compromising overall respiratory function (Figure 2D). Lung consolidation ensues due to the accumulation of inflammatory cells, fluid, and debris in the alveoli and bronchioles, resulting in added respiratory distress and in the appearance of a solid lung in imaging studies or during macroscopic gross examination. Interstitial pneumonia, another common pathological finding, also occurs due to inflammation and the thickening of the lung’s interstitial tissue, worsening the calf’s respiratory distress. In severe cases, the virus can cause bronchiolar obstruction and alveolar damage, compromising the calf’s overall respiratory function (Figure 2C,D) [63,64,65].

## 5. Host–Pathogen Interaction

Infection and replication are integral to the virus life cycle, but these processes expose viruses to detection and elimination by the host immune mechanisms. Viral nucleic acids, replication products, and some viral proteins are common detection targets of the innate immune system [66,67]. The timing and the kinetics of early viral detection by innate immune mechanisms significantly influence infection progression, the establishment of adaptive immunity, and ultimately impact disease outcome. Likewise, BRSV has evolved many escaping mechanisms to evade, delay, and/or subvert immune detection and antiviral host responses [68,69,70].

**Figure 3:**
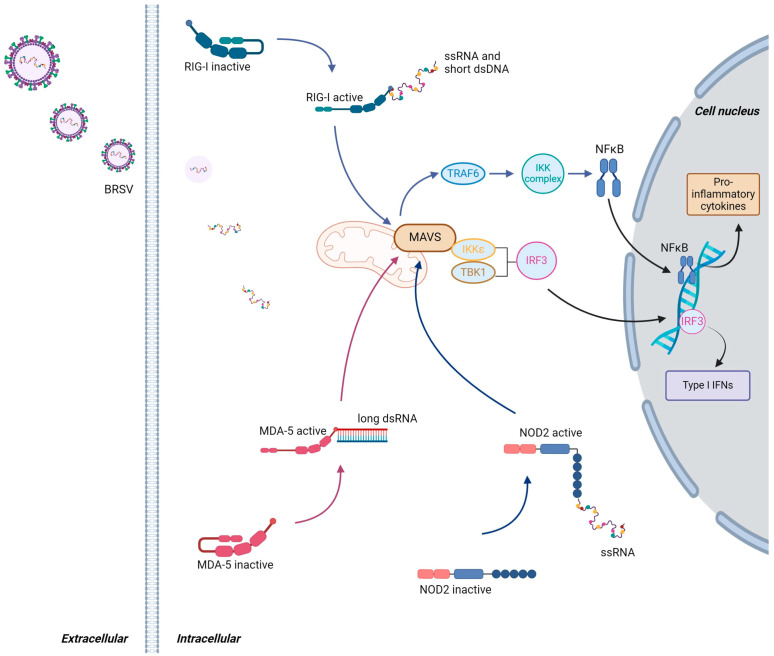
Schematic representation of the cytosolic sensing of BRSV by RIG-I, MDA-5, and NOD2. **Left:** BRSV binding and entry into the host cell, followed by the release of viral single-stranded RNA (ssRNA) into the cytoplasm. **Middle:** Viral RNA, originating both from the initial virion and from subsequent viral replication intermediates, is recognized by cytosolic sensors. RIG-I detects viral dsRNA and ssRNA with 5′-triphosphate groups; MDA-5 recognizes long dsRNA typically produced during viral replication; and NOD2 specifically senses ssRNA. Upon binding to their respective viral RNA ligands, RIG-I, MDA-5, and NOD2 undergo conformational changes, triggering downstream signaling pathways. All three sensors signal through the MAVS. MAVS then activates two key signaling cascades: the TRAF6-IKK and TBK1/IKKε pathways. TRAF6-IKK activation leads to NF-κB signaling, while TBK1/IKKε activation leads to the phosphorylation and activation of IRF3. **Right:** Once activated, NF-κB and IRF3 translocate to the nucleus, where they promote the transcription of pro-inflammatory cytokines and of type I IFNs, respectively, which play a crucial role in establishing an antiviral state. Abbreviations: BRSV = bovine respiratory syncytial virus; ssRNA = single-stranded RNA; dsRNA = double-stranded RNA; TRAF6 = tumor necrosis factor-alpha (TNF-α)-receptor-associated factor 6; NFκB = nuclear factor kappa-light-chain-enhancer of activated B-cells; IFNs = interferons; MAVS = mitochondrial antiviral-signaling protein; IKKε = inhibitor of nuclear factor kappa-B kinase ε; TBK1 = TANK binding kinase 1 (TBK1); IRF3 = interferon regulatory factor 3; NOD2 = nucleotide oligomerization domain 2; RIG-I = retinoic acid-inducible gene I; MDA-5 = melanoma differentiation-associated protein 5. Figure created using BioRender (BioRender.com/r20i079).

**Figure 4:**
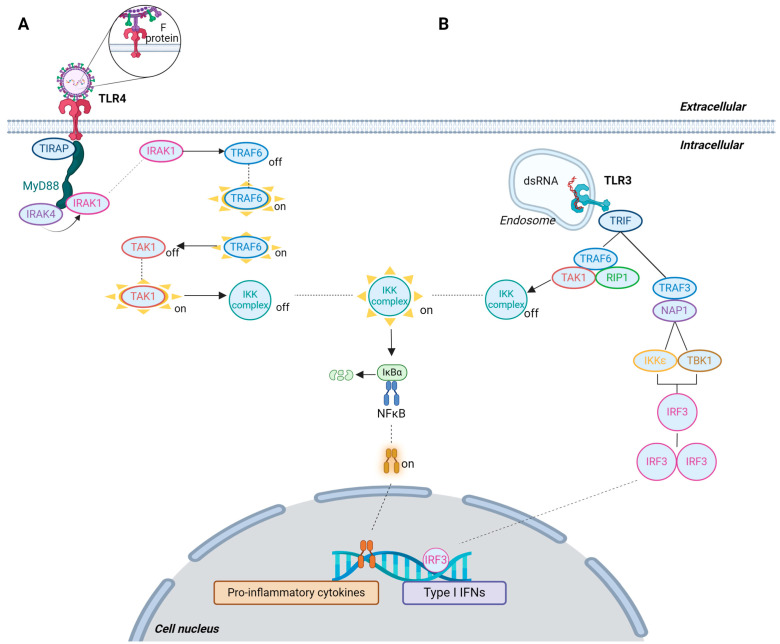
Schematic representation of endosomal and surface TLR signaling. (**A**) Recognition of F by TLR-4 culminates in NFκB activation and translocation to the nucleus, initiating the secretion of pro-inflammatory cytokines. (**B**) Recognition of dsRNA (a BRSV replication product) by TLR-3 leads to the activation of both NFκB and IRF3 and transcription of type I IFN and pro-inflammatory cytokines. Abbreviations: dsRNA = double-stranded RNA; TLR-3 and TLR-4 = toll-like receptor 3 and 4; IκBα = nuclear factor of kappa light polypeptide gene enhancer in B-cells inhibitor alpha; TRIF = toll interleukin 1 receptor (TIR)-domain-containing adaptor protein inducing interferon-beta; TRAF3 and TRAF6 = tumor necrosis factor-alpha (TNF-α)-receptor-associated factor 3 and 6; NAP1 = NFκB-activating kinase (NAK)-associated protein 1 (NAP1); NFκB = nuclear factor kappa-light-chain-enhancer of activated B-cells; TBK1 = TANK binding kinase 1 (TBK1); IRF3 = interferon regulatory factor 3; TAK1 = transforming growth factor beta (TGF-β)-activated kinase 1; RIP1 = receptor-interacting protein 1; F protein = fusion protein; TIRAP = toll/interleukin-1 receptor like-domain containing adaptor protein; MyD88 = myeloid differentiation primary-response 88; IRAK1 and IRAK4 = interleukin 1 receptor (IL-1R)-associated kinase 1 and 4; IFNs = interferons. Figure created using BioRender (BioRender.com/l09e767).

The host immune system is equipped with an arsenal of pattern recognition receptors (PRRs) that convert microbial and danger sensing into immediate host defense responses (Table 1 and Figure 3 and Figure 4). Viral detection results in a series of signaling pathways culminating in the secretion of type I interferons (IFNs), pro-inflammatory cytokines, and other inflammatory mediators, resulting in leukocyte recruitment, tissue infiltration, and later on, T-cell activation and antibody production. While these are steps necessary to achieve viral clearance, those responses frequently contribute to clinical signs, including fever, cough, increased mucus production, anorexia, respiratory distress, and depression [71,72,73].

### 5.1. Innate Immunity

Initial BRSV infection and replication occur in the superficial layer of the nasal, tracheal, and bronchial epithelium, and later replication occurs in the bronchiolar and alveolar epithelium. The host innate detection is based on the presence of PRRs, which sense distinct evolutionarily conserved structures on pathogens, termed microbial-associated molecular patterns (MAMPs). PRRs include toll-like receptors (TLRs), retinoic acid-inducible gene I-like receptors (RLRs), nucleotide oligomerization domain-like receptors (NLRs), and cytosolic DNA sensors. PRRs are expressed in cells of the immune system as well as non-immune cells and are present in multiple cellular compartments (cytosol, endosomal, or cell membrane), allowing virally infected cells to trigger defense mechanisms that decrease replication and signal infection to neighboring cells, triggering innate and adaptive immunity components [74,75]. In response to viral MAMPs’ detection, cells produce type I IFNs, particularly IFN-α and IFN-β, which are crucial in inhibiting viral replication and spread. These IFNs signaling in neighboring cells activate the Janus kinase/signal transducers and activators of transcription (JAK-STAT) signaling pathways, leading to the expression of hundreds of genes with antiviral functions [76,77].

While some viral proteins can trigger PRRs, nucleic acids detected in intracellular spaces during replication are predominant viral molecular patterns. Upon viral entry into host target cells, the BRSV viral RNA and its replication products are recognized by the nucleotide oligomerization domain 2 (NOD2), retinoic acid-inducible gene I (RIG-I), and melanoma differentiation-associated protein 5 (MDA-5), leading to the production of type I IFNs and establishing an antiviral state within the infected and neighboring cells. In this state, various defense mechanisms are upregulated, including the expression of antiviral proteins, to thwart viral replication and spread. This intricate interplay between cytosolic detection and the establishment of an antiviral state is a crucial aspect of the innate immune system’s ability to combat viral infections effectively [77,78,79]. In addition to type I IFN, innate immune cells release pro-inflammatory cytokines and mediators, such as interleukin-1 (IL-1), interleukin-6 (IL-6), and tumor necrosis factor-α (TNF-α), which contribute to inflammation and the recruitment of immune cells to the site of infection, while also mediating systemic clinical signs associated with the infection.

While the NOD2 and RIG-I are the most important NLR and RLR involved in the immune response against BRSV, the heterodimer TLR-2-TLR-6, TLR-3, TLR-4, TLR-7, and TLR-8 have also been shown to recognize BRSV [80,81,82,83,84]. It is essential to highlight that the cellular expression of PRRs, as well as the levels of their expression, differs among different cell types and stimuli, as do the signaling molecules involved in the pathway that leads to cytokine and IFN production. For example, dendritic cells (DCs) are specialized in expressing TLR-3, TLR-7, and TLR-9 with the activation of interferon regulatory factor 7 (IRF7), leading to a strong type I IFN secretion. These cells are in contrast with macrophages, known for their high expression of TLR-2 and TLR-4, primarily producing pro-inflammatory cytokines [74,85]. In cattle, γδ T-cells also express TLR-2, TLR-3, TLR-4, and TLR-7 [86]. Epithelial cells are crucial in the BRSV response, as they are not only a physical barrier but also the virus’s main target and, therefore, involved in immune activation. BRSV G protein binds to sialic acid residues on cell surfaces, and the F protein works with the G protein to mediate cellular invasion [22,87]. This virus has been shown to infect different respiratory tract cells in the trachea, bronchi, and pneumocytes [87,88]. The main PRRs involved in the anti-BRSV response, the cell types in which they are present, their location within the cell, and the MAMPs each one recognizes (their main ligands) are described in Table 1.

**Table 1:** Pattern recognition receptors putatively involved in the immune response against bovine respiratory syncytial virus.

PRR	Cell Types	Location	Ligand
TLR-2/6 [89]	Dendritic cells [90], macrophages [91], monocytes [91], neutrophils [92], mast cells [93], eosinophils [94], basophils [95], γδ T-cells [86], epithelial cells [96]	Cell surface [74]	Large attachment Glycoprotein (G) [80,97]
TLR-3 [98]	Dendritic cells [90,99] macrophages [100], eosinophils [94], mast cells [101], epithelial cells [96], γδ T- = cells [82]	Endosome [74]	Viral dsRNA [82,102]
TLR-4 [103]	Dendritic cells [90], macrophages [89], mast cells [93], neutrophils [92], monocytes [90], eosinophils [104], epithelial cells [105]	Cell surface [74]	Fusion protein (F) [43,80,81,83]
TLR-7 [90]	Dendritic cells [90], neutrophils [92], macrophages [89], eosinophils [104], epithelial cells [98], γδ T-cells [106]	Endosome [74]	Viral ssRNA [107,108]
TLR-8 [84]	Dendritic cells [90], macrophages [89], neutrophils [109], eosinophils [94], monocytes [110], regulatory T-cells [111], epithelial cells [112]	Endosome [74]	Viral ssRNA [107,108]
NOD2 [113]	Dendritic cells [114], macrophages [115], monocytes [116], epithelial cells [117]	Cytoplasm [118]	Viral ssRNA [118]
RIG-I [84]	Dendritic cells [119], macrophages [120], neutrophils [121], epithelial cells [122]	Cytoplasm [123]	Viral ssRNA [118]
MDA-5 [84]	Dendritic cells [124], macrophages [125], neutrophils [121], epithelial cells [126]	Cytoplasm [118]	Viral dsRNA [127]

Abbreviations: PRR = pattern recognition receptor; TLR-2/6, TLR-3, TLR-4, TLR-7, TLR-8 = toll-like receptor 2/6, 3, 4, 7, and 8; NOD2 = nucleotide oligomerization domain 2; RIG-I = retinoic acid-inducible gene 1; MDA-5 = melanoma differentiation-associated protein 5; ssRNA = single-stranded RNA; dsRNA = double-stranded RNA.

#### Pattern Recognition Receptors’ Signaling Pathways

NLRs and RLRs are key components of the cytosolic sensing system within cells. These receptors play a crucial role in the innate immune response, detecting MAMPs and damage-associated molecular patterns (DAMPs) within the cytosol of cells. NLRs and RLRs are involved in the immune response against BRSV (Figure 3). NOD2 detects viral ssRNA and is associated with mitochondrial antiviral-signaling protein (MAVS), also known as IFN-β promoter stimulator 1 (IPS-1), virus-induced signaling adaptor (VISA), or caspase activation recruitment domain-adaptor inducing IFN-β (CARDIF) [75]. After interaction with MAVS, transcription factor interferon regulatory factor 3 (IRF3) and nuclear factor kappa-light-chain-enhancer of activated B-cells (NFκB) are activated and lead to type I IFNs and pro-inflammatory cytokines’ production, respectively [128].

RIG-I and MDA-5 have been shown to detect RSV, with some evidence in cattle (BRSV) [10,78,129,130]. RIG-I is activated by 5′ triphosphorylated ends of RNA and uses MAVS as an intermediate. The pathway culminates in IRF3 and NFκB activation and the secretion of type I IFN and pro-inflammatory cytokines [131,132]. RIG-I activity with RSV recognition has been shown in cell lines and primary cell cultures [133,134,135], as well as in vivo in mice [136], human patients hospitalized due to RSV [137], and in BRSV-infected calves [130].

The sensing of cytosolic dsRNA by MDA-5 also recruits the adaptor protein MAVS, leading to the activation of TANK-binding kinase 1 (TBK1) and the inhibitor of nuclear factor kappa-B kinase ε (IKKε) [138]. TBK1 and IKKε induce IRF3 phosphorylation, which culminates with its translocation to the nucleus, where IRF3 induces type I IFNs’ production [75]. According to Loo et al. [139], RIG-I, but not MDA-5, was crucial in the immune defense against RSV, and cells lacking RIG-I were more permissive to RSV infection than wild-type cells and cells lacking MDA-5. MDA-5 may also be involved in the RSV modulation of type I IFN, as it colocalizes with the N protein inside inclusion bodies early post-infection [134].

TLRs are structurally composed of leucine-rich repeats (LRRs), which are responsible for ligand recognition, and a toll/interleukin-1 receptor-like (TIR) domain. The TIR domain is responsible for interacting with adaptor proteins to engage the signaling process [140]. Generally, every TLR uses the myeloid differentiation primary response 88 (MyD88) signaling pathway, except for TLR-3, which uses only TIR-domain-containing adapter-inducing interferon-β (TRIF), also known as TIR-containing adapter molecule 1 (TICAM-1), as an adaptor protein, and TLR-4, which can use both MyD88 and TRIF [141,142].

TLR-2 forms a dimer with either TLR-1 or TLR-6; however, previous studies have shown that especially the RSV species are recognized by the dimer TLR-2-TLR-6, and not TLR-2-TLR-1 [143], and that the TLR2-TLR6 dimer recognizes RSV G protein [97]. The signaling pathway executed by TLR-2 uses TIRAP (toll/interleukin-1 receptor like-domain containing adaptor protein), also known as MyD88-adaptor-like (MAL), as the adaptor to MyD88, leading to NFκB translocation and pro-inflammatory cytokines’ production [144]. TLR-4 was the first TLR reported to recognize viral pathogens and bind to the F protein of RSV [42] and BRSV [83]. Also, TLR-4 polymorphism is related to human RSV disease severity [145]. The TLR-4 pathway recruits either MyD88 or TRIF, and these pathways may be competitive (Figure 4A) [146]. TIRAP-MyD88 regulates early NFκB activation and the production of pro-inflammatory cytokines, while the TRIF-dependent pathway involves the recruitment of the adaptor proteins TRIF and TRIF-related adaptor molecule (TRAM). TRAM-TRIF signals activate IRF3 via TRAF3. IRF3 activation induces the production of type I IFNs [147,148,149]. On the MyD88-dependent pathway, once the TLR-4 recognizes the BRSV F protein (Figure 4A), the intracellular domain of the TLR recruits MyD88, which attracts interleukin 1 receptor (IL-1R)-associated kinase 1 (IRAK1) and 4 (IRAK4), forming a complex known as myddosome [150]. This formation activates IRAK4, which phosphorylates IRAK1, subsequently releasing IRAK1, which associates itself with TNF-α-receptor-associated factor 6 (TRAF6), activating it. The activated TRAF6 forms a new complex, along with transforming growth factor beta (TGF-β)-activated kinase 1 (TAK1), which is now activated. TAK1 then activates the IKK complex, which is composed of IKKα, IKKβ, and IKKγ, also known as NFκB essential modulator (NEMO). Once activated, the IKK complex phosphorylates the inhibitor of NFκB, which can now translocate to the cell nucleus, where it leads to the transcription of critical pro-inflammatory cytokines such as IL-1, IL-6, and TNF-α [74,85,151].

The secondary route executed by TLR-4 uses TRIF in place of MyD88 and leads to the increased production of type I IFNs [147,152]. The MyD88-independent pathway executed by TLR-4 is similar to endosomal TLR-3 signaling, shown in Figure 4B. Upon TRIF adaptor protein recruitment, TRAF6 is engaged, which interacts with both TRAF3 and receptor-interacting protein 1 (RIP1). TRAF6 is involved in NFκB activation by forming a complex with RIP1 and TAK1 which activates the IKK complex, phosphorylating IKκA, which in turn phosphorylates the NFκB inhibitor nuclear factor of kappa light polypeptide gene enhancer in B-cells inhibitor alpha (IκBα). Upon phosphorylation, IκBα dissociates from NFκB, undergoing ubiquitination and degradation. Once IκBα dissociates from the complex, NFκB can translocate to the nucleus, where it will induce the production of inflammatory cytokines (Figure 4) [85,153,154]. Alternatively, TRIF recruits TRAF3, which binds to NFκB-activating kinase (NAK)-associated protein 1 (NAP1), attracting TANK binding kinase 1 (TBK1) and IKKε, forming a complex. TBK1 and IKKε are responsible for phosphorylating IRF3. Phosphorylated IRF3 forms dimers that are the active form of the transcription factor and translocate into the nucleus. Once in the nucleus, IRF3 dimers bind to specific DNA sequences known as interferon-stimulated response elements (ISREs) present in the promoters of target genes, inducing type I IFN production (IFN-α and IFN-β) [155]. Moreover, in response to TLR-3 activation by BRSV, γδ T-cells present higher levels of monocyte chemoattractant protein-1 (MCP-1, also known as C-C motif chemokine ligand 2, CCL2) [82]. MCP-1 directs leukocyte infiltration, affects T-cell proliferation and function, activates macrophages, and promotes inflammation. MCP-1 may also act as a regulator in the polarization of T-cells toward a Th2 phenotype [156,157,158].

Infection with BRSV leads to an increase in several cytokines and chemokines in circulation, including IL-6, TNF-α, IL-18, chemokine-x-c motif ligand (CXCL8), CCL2, CCL3, and CCL5, mediating the subsequent influx of leukocytes, predominantly neutrophils [159,160]. A whole blood transcriptome analysis in BRSV-infected dairy calves revealed that several genes in the Kyoto Encyclopedia of Genes and Genomes (KEGG) pathway for Influenza A were influenced by the infection, including bovine-MHC genes (BOLA-DQ5 and BOLA-DQB), chemokines and cytokines (CXCL8, IL-12B, CXCL10), viral detection genes (DDX58 and IFIH1, which encode RIG-I and MDA5, respectively), and genes for cellular antiviral response and innate immune signaling (MX1, IRF7, EIF2AK2, RSAD2, OAS1Y, and OAS1Z) [10,129,130].

BRSV-infected lungs show intense neutrophilic infiltration that coincides with the occurrence and peak of clinical signs [63,161]. NETosis is a specialized form of cell death performed by neutrophils characterized by the release of neutrophil extracellular traps (NETs). This process involves expulsing DNA, histones, and antimicrobial proteins from the neutrophil, forming a web-like structure that traps and neutralizes pathogens [162,163,164,165]. RSV stimulates NETosis, resulting in the release of reactive oxygen species (ROS) [166]. The mechanisms underlying NET formation require ROS production, and while both NETosis and ROS are important immune mechanisms, exacerbated responses damage tissue. ROS and NETosis have been implicated in lower respiratory tract disease pathogenesis caused by BRSV and RSV [167,168,169]. The results of a study using a bronchoalveolar lavage to sample BRSV-infected calves are also consistent with neutrophil activation and NETosis and decreased antioxidant repairing mechanisms that would counteract tissue damage [168].

Oxidative stress, an imbalance in the production of ROS and antioxidant responses, was also reported in vitro in a BRSV infection of bovine turbinate primary cell line and ex vivo in lung tissues with lesions versus without lesions of BRSV-infected calves, with increased ROS genes and cyclooxygenase (COX)-2 (infected vs. non-infected) and decreased myeloperoxidase (MPO), NADPH oxidase (NOX)1, NOX4, and NOX5), and superoxide dismutase 1 (SOD-1) (lesioned vs. non-lesioned lungs of infected animals) [170]. SOD-1 products are anti-oxidative and mediate the removal of oxygen radicals. The evaluation of global changes in mRNA abundance in healthy lung and lung lesions from BRSV-infected calves also pointed to oxidative stress and the production of nitric oxide and ROS to be activated in the tissues of the BRSV-challenged animals in another study [171].

While nitric oxide and reactive oxygen are immune mechanisms involved in pathogen destruction and apoptosis, they have been shown to be used by viruses to facilitate replication [172,173,174]. Supporting the idea that the virus may benefit from intense lung neutrophilic infiltration and oxidative response, the differential regulation of cytokine production by IL-17A and IL-17F (which support neutrophils function and downregulate IL-10, an important anti-inflammatory cytokine) has also been reported in lung lesions [171]). Collectively, a transcriptome analysis of lung tissues with lesions versus without lesions from calves infected with BRSV supports dysregulated oxidative stress, cell damage, and depressed innate and adaptive immune functions [171,175]. The downregulation of B- and T-cells’ activation and differentiation, cytokine production, and adaptive immune response were typical findings in lesioned tissue samples, while the chemotaxis of immune cells (particularly innate immune cells) was increased. Differential gene expression suggested that increased chemotaxis may be a compensatory mechanism to the depressed functional activity of innate immune cells in lesions and their overall inability to efficiently control the infection.

Limiting inflammation and immune cells’ influx into the lung therapeutically by the use of broad-spectrum antioxidants and nonsteroidal anti-inflammatory drugs (NSAIDs), including COX-2 inhibitors, was attempted to curb tissue damage and improve lung function and disease outcome in humans [176]. While in mice, the use of such drugs has been shown to be promising, in calves, the treatment of BRSV-infected calves with ibuprofen (an NSAID) was correlated with increased viral shedding, even though it reduced weight loss and clinical illness [177]. In contrast, the use of ibuprofen as an adjunct therapy with antiviral fusion protein inhibitor (FPI) was shown to enhance the specific antiviral effect of FPI and reduce the damaging impact of prostanoids and oxidative stress [62,175,178].

### 5.2. Adaptive Immunity

BRSV may cause exacerbated respiratory tract inflammation and the recruitment of innate leukocytes to the lungs of infected animals, accompanied by decreased cell function and tissue-sparing mechanisms [170,171,175,179]. Adaptive immunity is initiated by the action of professional antigen-presenting cells (APCs) that carry foreign material from the infection site into secondary lymphoid organs to activate T-cells, while the lymph drainage of infected sites carries native antigens for B-cell recognition and activation [180]. Innate defense events may impact the ability of DCs to properly and timely activate protective adaptive responses.

BRSV is most severe in young animals, which present inexperienced immune systems and a concomitant downregulation of several immune mechanisms [181]. Immunosuppression in neonates is highly dynamic and tightly regulated, with a short and early stage when T-cell subsets synergize towards a narrow pro-tolerogenic window that favors non-inflammatory colonization [181,182]. BRSV infection in neonates may also occur during the pulmonary transition to life after birth [183,184,185]. Significant physiological changes in respiratory and hemodynamic function are initiated by breathing at birth and the clamping of the umbilical cord, and a complex adaptation takes place several weeks after birth [186,187,188]. While those processes contribute to the maturation of the lungs and the immune system, they hamper effective immune responses against BRSV [182,184,185]. Additionally, BRSV employs different strategies to curb innate and adaptive immune responses, which have detrimental effects on immunological memory [5,189]. DC function may be subverted during infection, leading to imbalanced adaptive immune responses: more protective responses, such as T helper (Th) 1 activity being delayed or suppressed (low IL-12 production) and harmful responses (Th2, with some evidence of Th17) being favored (IL-4, IL-10, IL-33, IL-13, IL-22, IL-6, and TGF-β) [190,191]. T-cells are important effector immune cells but are only activated after a highly orchestrated differentiation process. Upon an initial encounter with DCs in secondary lymphoid organs, naïve T-cells integrate signals through their T-cell receptor (TCR) and co-stimulatory molecule interactions [192]. This initial priming is amplified by cytokine signaling, for example, IL-12 differentiates CD4 T-cells into their Th1 phenotypes [193,194], and IL-12 and type I IFN activate naïve CD8 T-cells in their cytotoxic profile [195,196]. IL-4 secretion differentiates CD4 T-cells into their Th2 phenotype, while IL-6 and TGF-β production together leads to Th17 differentiation. Additionally, paracrine and autocrine IL-2 signaling allows T-cells to initiate a program of rapid and extensive division and differentiation [197,198,199].

Detrimental responses to BRSV have been more extensively studied in the context of vaccine-associated enhanced disease (VAERD). Both RSV and BRSV formalin-inactivated (FI) vaccination have resulted in enhanced respiratory disease after virulent virus infection [200,201,202,203,204]. Th2-biased responses are frequently associated with FI-BRSV vaccines [200,202] and have also been observed in the context of BRSV infection [205]. FI-BRSV vaccination in calves induces IL-4 production, high IgE titers [200,202], a significant influx of eosinophils in the lungs [200], and reduced IFN-γ production by lymphocytes [203]. Th2 responses trigger the activation of mast cells, eosinophils, and IgE-producing plasma cells [206], leading to inflammation and low levels of effectiveness against viral pathogens [204]. In fact, Th2 polarization, with IL-4 production and the presence of anti-BRSV IgE antibodies, is shown to correlate to disease severity and poor outcomes in cattle [73,205].

The development of an adaptive immune response, especially Th1 responses, is associated with BRSV infection control, and the clearance of BRSV is concomitant with the induction of BRSV-specific CD8 T-cell responses in peripheral blood and their influx into the lungs [207,208,209]. In cattle, the depletion of CD8 T-cells results in the delayed clearance of BRSV from the upper and lower respiratory tract and a more severe pulmonary pathology [207,209,210,211]. A bronchial lymph node transcriptome analysis in BRSV-infected calves revealed an intense activation of host immune response genes, with granzyme B being the most upregulated gene in the lymph nodes of infected calves in the study conducted by Johnston et al. [10]. Granzyme B has also been shown to be increased in CD8 T-cells, NK cells, and CD4 T-cells in tracheal aspirates from children infected with RSV [212].

Similar to the results of a whole blood transcriptome analysis [130], in the bronchial lymph node transcriptome, the KEGG pathway for Influenza A was also enriched, and upregulated genes, including cytokines responsible for the induction of the febrile response, programmed cell death, and interferon-stimulated genes [10], are also supported by the study of Tizioto et al. [81]. No indication of Th2 polarization was observed in these studies, although genes related to B-cell receptor signaling were consistently enriched. Likewise, IL-12, involved in Th1 polarization, was not detected as a differentially expressed gene, even though acute phase response signaling was upregulated [10,81]. Nevertheless, it is worth noting that calves in these studies presented mild to subclinical infections, and samples were obtained at a single time point (day 7 post-infection).

Features of severe BRSV infection in calves include rapid neutrophil infiltration, excessive mucus production, delayed virus-adaptive T-cell response [161], and the expression of Th2 or Th17 cytokines, such as IL-4, IL-13, and IL-17 [213,214,215]. The therapeutic inhibition of IL-17 by digoxin can limit BRSV-associated disease in neonatal calves without significant adverse impacts on the viral burden [213]. Regarding the recognition of RSV proteins, T-cell responses are directed at epitopes within several viral proteins, including N, M, NS2, M2-1, F, and G proteins [216]. The F and G proteins are major antigenic CD4 T-cell targets in humans and cattle [217,218]. The F protein of RSV and BRSV has been studied in depth and is described as containing multiple antigenic regions [55,71,219].

Mucosal immunity is crucial for protection against BRSV in limiting virus replication at the initial site of infection [71,220,221]. Studies in calves in which the primary mucosal response to BRSV was inhibited by maternally acquired antibodies (MDAs) and studies of BRSV vaccine efficacy suggest that the ability to mount a rapid secondary mucosal immunoglobulin (IgA) response may be more important in protection than the level of pre-existing mucosal antibodies [71,179,221,222,223]. There is also evidence that while the presence of MDA leads to poor antibody response to vaccination, memory cell formation is preserved, and vaccines may still elicit protective responses to subsequent challenges with rapid antibody production and T-cell responses, specific IgG, and neutralizing antibodies [224].

The persistence of memory T-cells post-infection ensures a more rapid and efficient response upon re-exposure to the virus, although BRSV usually leads to suboptimal memory responses [5,71]. Despite their multiple protective mechanisms, cattle, similar to RSV in humans, still experience multiple BRSV reinfections. Subsequent infections tend to be less severe but maintain the virus circulation in the population, contributing to the infection of highly susceptible young calves.

While adaptive immunity is essential for viral clearance, these responses may be delayed by the many evasion molecules employed by the virus, physiological immune regulation in neonatal calves, and the inexperienced immune system of young calves. Likewise, antibodies and T-cells have also been shown to contribute to BRSV pathogenesis and may participate in pathology, especially Th2 cells that support the production of BRSV-specific IgE antibodies and eosinophil involvement in the context of formalin-inactivated vaccines [225,226].

## 6. Viral Immune-Evading Mechanisms

**Figure 5:**
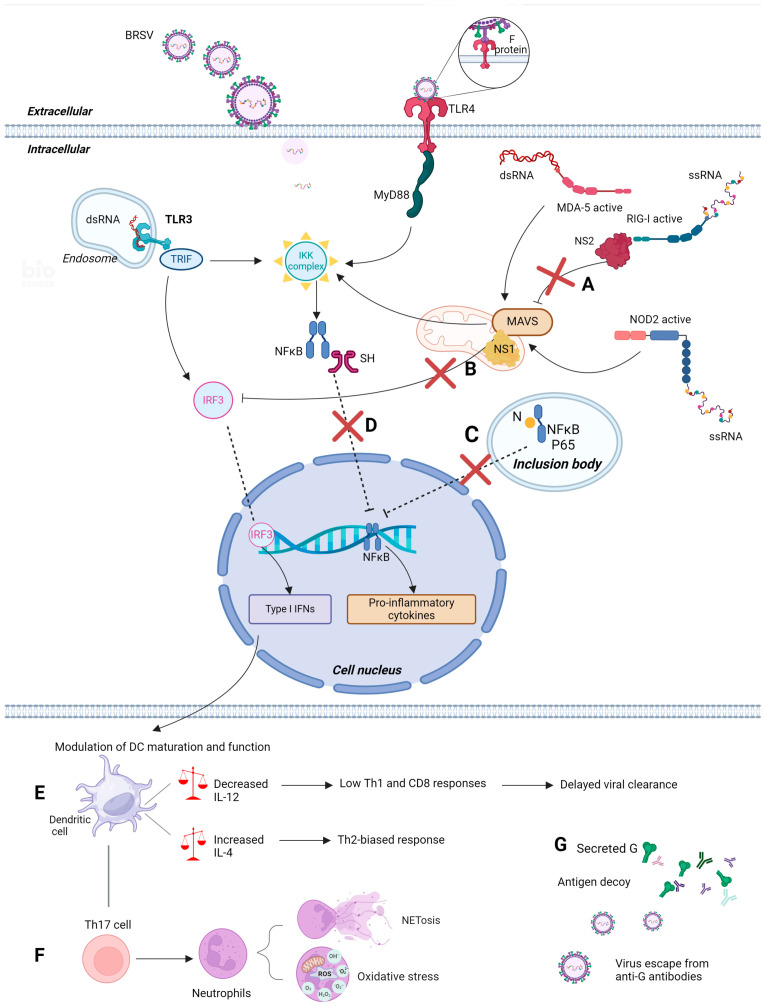
Schematic representation of some of the respiratory syncytial virus’s immune-evading mechanisms. (**A**) NS2 interacts with the CARD domain of RIG-I; (**B**) NS1 binds to MAVS, the adaptor protein used by the RIG-I receptor on the signaling pathway. These interventions inhibit IRF3 activation, preventing downstream pathway events and hindering type I IFN production. (**C**) N protein is involved in sequestering the P65 subunit of NFκB into intracytoplasmic inclusion bodies, leading to decreased translocation to the nucleus; (**D**) The SH protein interferes with pro-inflammatory cytokines’ production through the NFκB pathway. (**E**) Interference with IRF3 and NFκB pathways modulates the function of DCs and may suppress their maturation, resulting in decreased IL-12, which is essential for CD8 T-cell differentiation and viral clearance, and increased IL-4, leading to poorly protective and potentially detrimental Th2-biased responses. (**F**) BRSV infection may induce Th17 differentiation, contributing to neutrophil accumulation, NETosis, and oxidative stress, which are thought to enhance pathology. (**G**) BRSV-secreted G protein (sG) acts as a decoy antigen, sequestering antibodies away from the actual virion. Abbreviations: BRSV = bovine respiratory syncytial virus; dsRNA = double-stranded RNA; ssRNA = single-stranded RNA; TLR-3 and TLR-4 = toll-like receptor 3 and 4; TRIF = toll interleukin 1 receptor (TIR)-domain-containing adaptor protein inducing interferon-beta; IRF3 = interferon regulatory factor 3; NFκB = nuclear factor kappa-light-chain-enhancer of activated B-cells; IL-4 and IL-12 = interleukin 4 and 12 F protein = fusion protein; SH = small hydrophobic protein; NS1 and 2 = nonstructural proteins 1 and 2; G = major attachment glycoprotein; N = nucleoprotein; MyD88 = myeloid differentiation primary-response 88; MAVS = mitochondrial antiviral-signaling protein; NOD2 = nucleotide oligomerization domain 2; RIG-I = retinoic acid-inducible gene I; MDA-5 = melanoma differentiation-associated protein 5; Th1, Th2 and Th17 = T helper cells 1, 2 and 17; IFNs = interferons. Figure created using BioRender (BioRender.com/e06u568).

BRSV has evolved several mechanisms for evading the immune response to facilitate prolonged virus replication in the host. Two nonstructural proteins, NS1 and NS2, are the first to be produced and, therefore, are abundant in early infection (Figure 2A) [22,227]. Recombinant BRSV lacking NS1/NS2 proteins induced higher type I IFN levels in bovine cell cultures when compared to wild-type BRSV, and the viral replication was significantly higher in 2-year-old calves challenged with wild-type BRSV than in those that received the recombinant BRSV lacking NS proteins [228]. Moreover, NS1 and NS2 can work independently and together to suppress IFN production [229,230]. NS1 binds to MAVS, the adaptor protein that the RIG-I receptor uses in the signaling pathway, and NS2 interacts with the CARD domain of RIG-I. These interventions inhibit IRF3 activation, preventing downstream signaling from happening (Figure 5A,B) [230,231,232,233,234]. Interference by NS proteins in the IRF3 pathway has been described for both RSV and BRSV [230,232,234]. Additionally, the G protein from RSV was shown to inhibit IFN-stimulated genes (ISGs) in epithelial cells from mice [235] and humans [236].

Inclusion bodies perform multiple roles in the virus’s life cycle, including the compartmentalization of viral replication and transcription (Figure 2A). Recently, BRSV inclusion bodies were shown to participate in evasion mechanisms by sequestering cellular proteins involved in the antiviral response. In BRSV-infected cells, NFκB component p65 is recruited to inclusion bodies during viral replication (Figure 5C), blocking NFκB translocation to the nucleus and the transactivation of the NFκB reporter, even after TNF-α stimulation. While inclusion bodies were shown to contain BRSV N, P, L, M, and M2-1 proteins, light electron microscopy indicated colocalization, pointing to a possible interaction between N and p65 in BRSV inclusion bodies [70]. The SH protein was also shown to prevent apoptosis in infected cells in vitro and to interfere with pro-inflammatory cytokine production through NFκB pathway inhibition (Figure 5D) [237,238].

By interfering with antiviral mechanisms, BRSV infection also suppresses the maturation of DCs, which can all be, at least partially, a result of type I IFN inhibition (Figure 5E) [228,239,240,241]. The interference of the type I IFN pathway is so significant that type III IFN may predominate in RSV antiviral responses, differing from most other viruses [135]. Cytotoxic T-cells and Th1 cells become activated by the IL-12 secreted by DCs [242,243,244]. However, the modulation of DC activity by BRSV inhibits IL-12 secretion and may favor the release of interleukin-4 (IL-4). The lack of IL-12 and the presence of IL-4 result in a Th2-biased response (Figure 5E) [245]. Other studies have shown that human RSV interaction with DCs reduces their ability to produce essential cytokines that would lead to an effective T-cell response [66], and further research on BRSV described similar findings in experimentally infected calves [246]. In this scenario, protective BRSV-specific CD8 T-cell responses are suppressed or delayed [66,247]. This Th2-biased response, that is not only induced by NS proteins, is correlated with disease severity, as the clinical signs are not necessarily caused by the virus alone but also by the immune response to the infection.

The N protein can also inhibit T-cell activation in RSV infection [248]. In mice, NS1 was shown to reduce IL-17 production [249] and to decrease viral load in the lungs [250]. However, Long et al. [251] reported an association between increased IL-17 levels and the severity of RSV-caused pneumonia in mice. BRSV-infected calves also express significant levels of IL-17 (primarily produced by activated γδ and Th17 cells) [214,252]. In vitro analyses have also shown high levels of IL-17 production after BRSV infection and even higher levels when peripheral blood mononuclear cells (PBMCs) are co-infected with *Mannheimia haemolytica*, another important agent involved in BRD [214]. In neonatal BRSV-infected calves, reduced IL-17 levels lead to improved disease outcomes and milder disease. Nevertheless, BRSV infection was shown to lead to Th17 cell differentiation [214]. The consequent IL-17 production recruits neutrophils [253], leading to NETosis and ROS-formation-enhancing pathology (Figure 5F) [166,169].

In addition to being produced in its full length as a transmembrane protein, the RSV G sequence contains a second translation initiative site that leads to the production of a truncated G form, which is secreted (sG). This is preserved in RSV and BRSV viruses and has been suggested as an evading mechanism (Figure 5G) [254]. In addition, the G protein participates in the Th2-biased induced response, as it enhances IL-4 levels, which leads to Th2 production and suppresses the Th1 transcription factor [255]. The F protein, despite acting mainly to incite immune response and not to evade it, can cause damage to the host, as the F-TLR-4 signaling pathway induces the formation of NETs [256]. NETs have been shown to provoke extensive injury to the respiratory tract during viral infections, with reports in cattle [62]. Considering all these factors, BRSV has the potential to evade the host immune defenses and succeed in replicating and spreading further, emphasizing the need for greater vaccines against this agent.

## 7. Conclusions

In summary, many viral proteins play a role in delaying the translation of viral detection into effective immune responses. The inhibition of type I IFN and establishment of an anti-viral state paradoxically occur with the abundant recruitment of phagocytes that poorly control viral replication and spread. The resulting adaptive response, in the context of decreased Th1 polarizing cytokines, may result in Th2- or Th17-biased responses, which further allow for viral replication, phagocyte recruitment, and inflammation, aggravating the clinical disease. Likewise, the virus may benefit from the induction of poorly protective and sometimes harmful adaptive immune responses to the detriment of cytotoxic T-cells and supporting Th1 responses that would halt viral replication and control inflammation. The present study reviewed the main PRRs involved in the anti-BRSV response, which MAMP they recognize, and the further signaling pathway initiated by viral recognition. Innate immunological components, such as IFN signaling and cytokine release, play a critical role in adaptive response, memory immunity, and disease outcomes. This paper also presents the escape mechanisms developed by this virus that may contribute to disease severity and impair lasting immunity development, contributing to maintaining the virus’s circulation and its high morbidity levels. Finally, a deeper and updated understanding of the immunological features involved in BRSV infection is essential for improving the current methods of prophylaxis and treatment.
